# Cinnamic acid alleviates endothelial dysfunction and oxidative stress by targeting PPARδ in obesity and diabetes

**DOI:** 10.1186/s13020-025-01064-7

**Published:** 2025-01-24

**Authors:** Yizhen Bai, Dechao Tan, Qiaowen Deng, Lingchao Miao, Yuehan Wang, Yan Zhou, Yifan Yang, Shengpeng Wang, Chi Teng Vong, Wai San Cheang

**Affiliations:** 1 State Key Laboratory of Quality Research in Chinese Medicine, Institute of Chinese Medical Sciences, University of Macau, Macao SAR, China; 2 Macau Centre for Research and Development in Chinese Medicine, University of Macau, Macao SAR, China

**Keywords:** Cinnamic acid, Endothelial function, Oxidative stress, PPARδ, Diabetes, Obesity

## Abstract

**Objective:**

Cinnamic acid (CA) is a bioactive compound isolated from cinnamon. It has been demonstrated to ameliorate inflammation and metabolic diseases, which are associated with endothelial dysfunction. This study was aimed to study the potential protective effects of CA against diabetes-associated endothelial dysfunction and its underlying mechanisms.

**Methods:**

High-fat diet (HFD) with 60 kcal% fat was used to induce obesity/diabetes in C57BL/6 mice for 12 weeks. These diet-induced obese (DIO) mice were orally administered with CA at 20 or 40 mg/kg/day, pioglitazone (PIO) at 20 mg/kg/day or same volume of vehicle during the last 4 weeks. Isolated mouse aortic segments and primary culture rat aortic endothelial cells (RAECs) were induced with high glucose (HG) to mimic hyperglycemia and co-treated with different concentrations of CA.

**Results:**

In DIO mice, four-week administration of CA, particularly at 40 mg/kg/day, diminished the body weights, blood pressure, fasting blood glucose and plasma lipid levels, and ameliorated endothelium-dependent relaxations (EDRs) and oxidative stress in aortas. The beneficial effects of CA were comparable to the positive control group, PIO. Western blotting results indicated that CA treatment upregulated the expression of peroxisome proliferator-activated receptor delta (PPARδ), and activated nuclear factor erythroid 2-related factor 2 (Nrf2)/ heme oxygenase-1 (HO-1) and AMP-activated protein kinase (AMPK)/ protein kinase B (Akt)/ endothelial nitric oxide synthase (eNOS) signaling pathways in mouse aortas in vivo and ex vivo. HG stimulation impaired EDRs in mouse aortas and inhibited nitric oxide (NO) production but elevated reactive oxygen species (ROS) levels in RAECs. CA reversed these impairments. Importantly, PPARδ antagonist GSK0660 abolished the vasoprotective effects of CA. Molecular docking analysis suggested a high likelihood of mutual binding between CA and PPARδ.

**Conclusion:**

CA protects against endothelial dysfunction and oxidative stress in diabetes and obesity by targeting PPARδ through Nrf2/HO-1 and Akt/eNOS signaling pathways.

## Introduction

Diabetes is a chronic metabolic disease characterized by chronic hyperglycemia and impaired carbohydrate, lipid, and protein metabolism. It is caused by complete or partial insufficiency of insulin secretion and/or insulin action [1]. Impairment of vascular endothelial function is a key indicator in the pathogenesis of cardiovascular complication in diabetes, making it a target of many anti-diabetic studies [2, 3].

In recent years, there has been a growing interest in investigating metabolic and cardiovascular benefits of natural products [4]. Among these, compelling evidence suggest that dietary polyphenols have emerged as a promising class of compounds for the prevention and management of diabetes and its complications [5]. One such polyphenol is cinnamic acid (CA), an organic acid found naturally in various plants, including cinnamon and benzoin [6]. CA (chemical formula: C_9_H_8_O_2_) features a typical benzene ring and carboxyl structure, serving as a key intermediate in the biosynthesis of various natural products within organisms [7]. It exhibits low toxicity [8] and is extensively utilized in the market for applications such as essence or spice production, fruit preservation, and other purposes [9]. Recent data have supported its beneficial effects such as antioxidant [10], anti-inflammatory [11], and potential anti-cancer properties [12]. Furthermore, its impact on diabetes has been demonstrated, such as enhancing insulin secretion, increasing glucose uptake, and inhibiting fat production [5]. Up to date, there is no report on the possible protective effect of CA in endothelial dysfunction related to obesity and diabetes. Notably, previous studies have reported a potential correlation between peroxisomes proliferator-activated receptors (PPARs) and CA [13, 14].

A large number of studies have revealed that activated nuclear factor erythroid 2-related factor 2 (Nrf2)/ heme oxygenase-1 (HO-1) and AMP-activated protein kinase (AMPK)/ protein kinase B (Akt)/ endothelial nitric oxide synthase (eNOS) signaling pathways are involved in regulating vascular homeostasis. Nitric oxide (NO) derived from eNOS is a major vasodilator [15]. Activation of AMPK and Akt can lead to phosphorylation and activation of eNOS to produce NO. In diabetes, AMPK/Akt/eNOS pathway in the endothelial cell is inhibited, which is related to endothelial dysfunction [16]. Oxidative stress is also an important contributor to endothelial dysfunction associated with diabetes and other diseases. Nrf2/HO-1 signaling pathway is one of crucial anti-oxidative mechanism and its activation can ameliorate hyperglycemia-induced endothelial dysfunction [17].

PPARs are a class of ligand-activated factors in the nuclear receptor superfamily, consisting of PPARα (NR1C1), PPARδ/β (NR1C2), and PPARγ (NR1C3) [18]. The PPAR family is widely involved in regulating the steady-state and protein expressions in various organs and tissues, and partakes different physiological processes including metabolism, immunity, growth and development [19, 20]. Extensive evidence from human and murine studies supports that PPARs play significant role in regulating vascular function [21]. PPARδ activation has been demonstrated to alleviate oxidative stress and endothelial dysfunction in diabetes [22].

This study was aimed to explore whether CA could offer protection against damaged vascular endothelium and oxidative stress in diabetes through modulation of PPAR isoform(s). By investigating the mechanisms involved, we provide the understanding of the potential use of CA for diabetic complication.

## Materials and methods

### Animal study and drug treatment

C57BL/6 male mice (6 weeks old) were randomly divided into 5 groups (6–8 mice per group). Mice in the control group (CON) were fed with normal rodent diet, while diet-induced obese (DIO) mice fed on a high-fat diet (60 kcal% fat; Changzhou SYSE Bio-tec Co., Ltd., China) for 12 weeks. At the last 4 weeks of diet, DIO mice of treatment groups were given with CA (20 and 40 mg/kg; Sigma-Aldrich, St. Louis, MO, USA) or pioglitazone (PIO; 20 mg/kg; Sigma-Aldrich) by oral gavage daily for 4 weeks, while mice in the CON and DIO groups were given with the same volume of vehicle. At the end of the study, mice were sacrificed, whose blood and aortas were collected for further study. The ethics of this animal protocol was approved on 31 August 2021 by the Animal Research Ethics Committee of University of Macau (protocol code UMARE-024-2021).

### Blood glucose levels and blood pressure measurement

Fasting blood glucose levels (FBG) were measured after fasting for 6 h (Yuwell Group, Jiangsu yuyue Medical Equipment & Supply Co., Ltd., China). Systolic (SBP) and diastolic (DBP) blood pressures were measured by a tail-cuff method using the CODA noninvasive blood pressure system (Kent Scientific Corporation, USA) in conscious mice.

### Determination of lipid parameters

After collecting plasma, the levels of total cholesterol, low-density lipoprotein (LDL) cholesterol, high-density lipoprotein (HDL) cholesterol, and triglycerides were measured by respective determination kits (Nanjing Jiancheng Bioengineering Institute, China). The OD values were detected by FlexStation 3 multifunctional microplate reader (Molecular Devices, Shanghai Co., Ltd., China).

### Ex vivo culture of mouse aortas

Mouse thoracic aortic segments were isolated in sterile PBS and subsequently incubated in Dulbecco’s modified Eagle’s medium (DMEM, low glucose; Gibco, Thermo Scientific Inc., USA) with 10% fetal bovine serum (FBS; Gibco) and 1% penicillin–streptomycin (P/S; Gibco) at 37 °C in a 5% CO_2_ environment. The aortas were treated with 30 mM glucose for 48 h to simulate a high-glucose (HG) environment, and some of them were co-treated with CA (10 or 30 μM). The same volume of mannitol was added to the control group (NG). Furthermore, co-incubation with PPARδ inhibitor (GSK0660; 5 μM; Sigma-Aldrich), PPARγ inhibitor (GW9662; 5 μM; Sigma-Aldrich), or PPARα inhibitor (GW6471; 5 μM; MedChemExpress, MCE, USA) was applied to evaluate the possible involvement of PPARs. These aortic segments were used for functional studies in wire myograph and protein determination by western blotting.

### Isometric force measurement in wire myograph

Arterial segments were suspended in a Multi Myograph System (Danish Myo Technology, Denmark) to determine the changes in isometric tension. Aortic segments from mice were stretched to optimal baseline tension (~ 3 mN), equilibrated for 60 min, and then given with 60 mM KCl to contract the aorta. Endothelium-dependent relaxations (EDRs) were studied in response to acetylcholine (ACh; 3 nM–10 μM; Sigma-Aldrich) upon pre-contraction of phenylephrine (Phe; 3 μM; Sigma-Aldrich) in endothelium-intact rings. In the presence of nitric oxide (NO) synthase inhibitor L-NAME, sodium nitroprusside (SNP; 1 nM–10 μM; Sigma-Aldrich)-induced endothelium-independent relaxations were subsequently measured. Each experiment was performed on rings prepared from different experimental mice.

### Primary culture of rat aortic endothelial cells (RAECs)

Type IA collagenase (2 mg/mL in PBS; Sigma-Aldrich) was used to digest rat aorta in a 37 °C water bath for 15 min. The suspension was centrifuged at 1800 rpm for 10 min to collect the detached endothelial cells. The cells were resuspended in RPMI 1640 culture medium (Gibco) with 10% FBS and 1% P/S and transferred to cell culture flask in a 37 °C incubator for 1 h to allow RAECs to adhere to the flask. Then, the culture medium was refreshed to remove unattached cells. RAECs were cultured in the flask until they reached ~ 90% confluence for passage and drug treatment. The cells were co-treated with 44.4 mM HG, CA (10 or 30 μM) and three inhibitors of different PPAR subtypes (5 μM) for 48 h. The treated cells and the cell culture supernatants were collected for further analysis. The accumulation of NO was detected by using Griess reagent (Beyotime Biotechnology, China) according to the manufacturer's instructions.

### Measurement of reactive oxygen species (ROS) production

Mouse aortic rings were frozen with Tissue-Tek O.C.T. Compound (Sakura Finetek, USA) and sliced into 10-µm thick sections under cold condition on cryostat. Aorta sections and treated RAECs were incubated with dihydroethidium (DHE; Thermo Scientific) at a final concentration of 5 μM diluted by normal physiological saline solution (NPSS, consisted of CaCl_2_, MgCl_2_, KCl, glucose, NaCl, and HEPES) for 30 min at 37 °C in the dark. DHE fluorescence was captured by Leica-DMi8 inverted fluorescent microscope (Danaher Corporation, USA) for aorta sections, and by Incucyte cell photography instrument (Bio-Rad Laboratories, USA) for RAECs. Image J software (USA) was used to analyze DHE fluorescence intensity.

### Western blotting

Mouse aortas or RAECs were lysed in ice-cold Radio Immunoprecipitation Assay Lysis buffer (RIPA, Beyotime Biotechnology) for 30 min to extract proteins. The protein concentrations were determined using an enhanced BCA protein assay reagent (Beyotime Biotechnology). The proteins were separated by 10–12% SDS-PAGE gel, and then were transferred to polyvinylidene fluoride (PVDF) membrane (Bio-Rad), blocked with 5% non-fat milk solution at room temperature for 60 min, and then incubated in primary antibodies (1:1000 dilution) at 4 °C overnight. Antibodies against activated nuclear factor erythroid 2-related factor 2 (Nrf2), heme oxygenase-1 (HO-1), phosphorylated protein kinase B (Akt) (Ser473), total Akt, total endothelial nitric oxide synthase (eNOS), and GAPDH were purchased from Cell Signaling Technology (USA). Antibodies against phosphorylated AMP-activated protein kinase α (AMPKα) (Thr172), total AMPKα, phosphorylated eNOS (Ser1177) were obtained from Proteintech Group (USA). Antibody against PPARδ was acquired from Abcam (UK). After incubation with HRP-labeled anti-rabbit or anti-mouse IgG secondary antibodies (Beyotime Biotechnology) at room temperature for 2 h, enhanced chemiluminescence (ECL) substrate (Thermo Scientific) was used to develop and image the membranes using the ChemiDoc MP Imaging System (Bio-Rad), and the data were analyzed by Image J software (USA).

### Molecular docking

Protein structures were obtained from Uniprot web, PDB web and literature comparisons (PDB code: 3TKM), and the solvent molecules and other impurities were removed by using PyMOL (USA). Then, PubChem was used to acquire the most accurate small molecule chemical structural formula. The pure molecules were docked by Schrödinger software (USA) and HOME for Researchers platform (China). Finally, the results were analyzed through Schrödinger’s computational platform (USA).

### Statistical analysis

Each experiment had at least four replicates. Graphpad Prism 8 software (USA) was used for data analysis and expressed as mean ± SEM. The data were analyzed using one-way ANOVA and Bonferroni post hoc tests. *P* < 0.05 was considered as statistically significant difference between groups.

## Results

### CA improved metabolic parameters in DIO mice

**Fig. 1:**
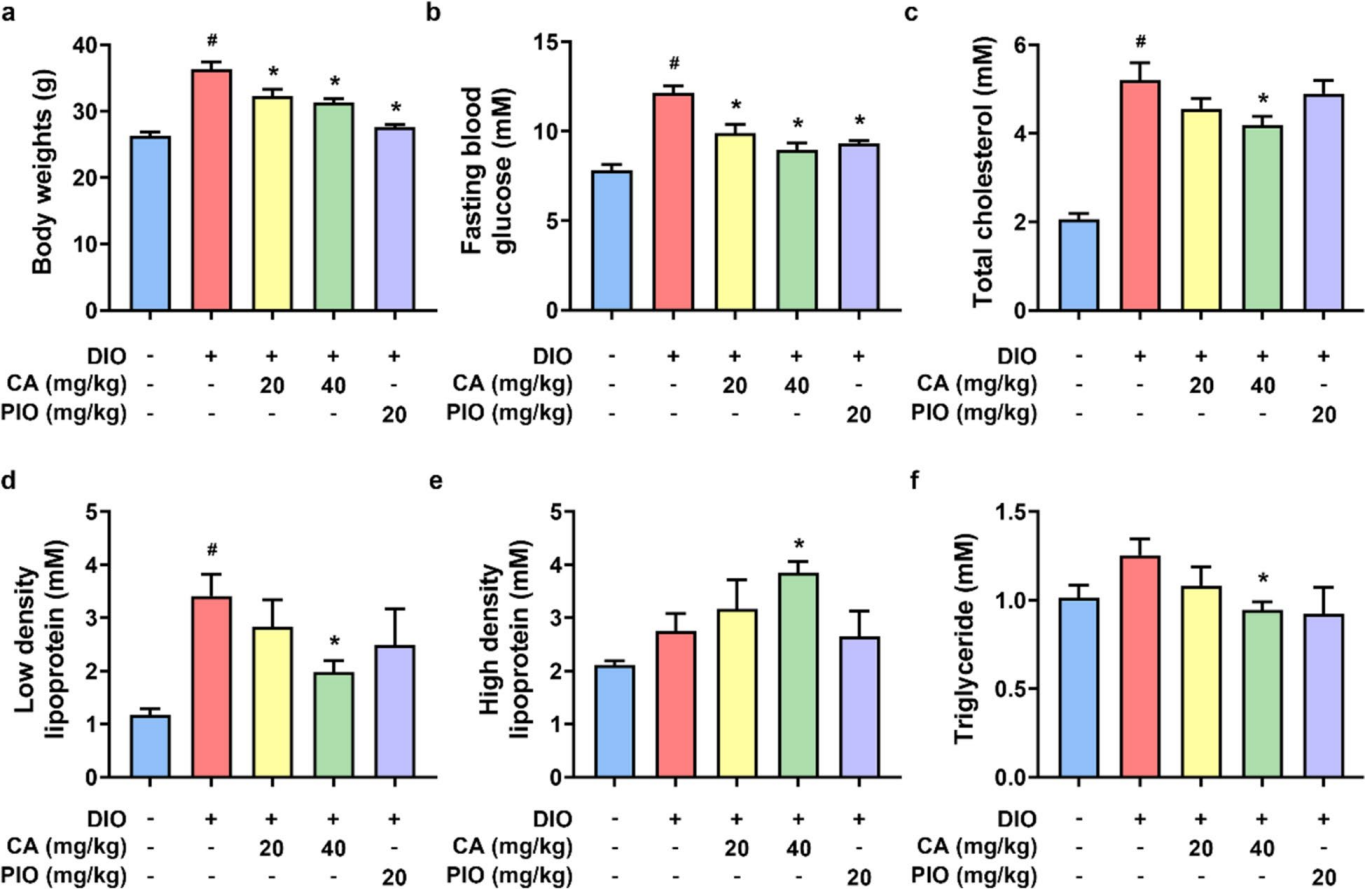
Effects of cinnamic acid (CA, 20 or 40 mg/kg/day, 4 weeks) and pioglitazone (PIO, 20 mg/kg/day, 4 weeks) on body weights, fasting blood glucose and cholesterol levels in diet-induced obese (DIO) mice (60 kcal% fat diet, 12 weeks). **a** Body weights; **b** fasting blood glucose levels; and the plasma levels of **c** total cholesterol; **d** low-density lipoprotein cholesterol; **e** high-density lipoprotein cholesterol; and **f** triglycerides. ^*#*^*P* < 0.05 vs. control (CON) group. ^*^P < 0.05 vs. DIO group. Data were shown as mean ± SEM (n = 6–8)

At the beginning of the experiments (week 0), mice of the same batch were randomly assigned into different groups and the body weights were similar among all groups (data not shown). At the end of the experiments (week 12), the DIO group exhibited a significant increase in body weight and FBG when compared with the CON group (Fig. 1a, b), indicating the successful establishment of the diabetic obese model. Four-week oral administration of CA and PIO alleviated these changes (Fig. 1a, b). We also measured the plasma levels of lipids, such as total cholesterol, LDL cholesterol, HDL cholesterol, and triglycerides (Fig. 1c–f). The treatment of CA, particularly at higher dosage of 40 mg/kg/day relieved the increase in total cholesterol, LDL cholesterol and triglycerides levels in DIO mice. In terms of the “good cholesterol” HDL cholesterol, we observed that CA increased its content in DIO mice. PIO group, positive control group, showed insignificant effects on plasma lipid levels.

### CA improved endothelial function and blood pressure in DIO mice

**Fig. 2:**
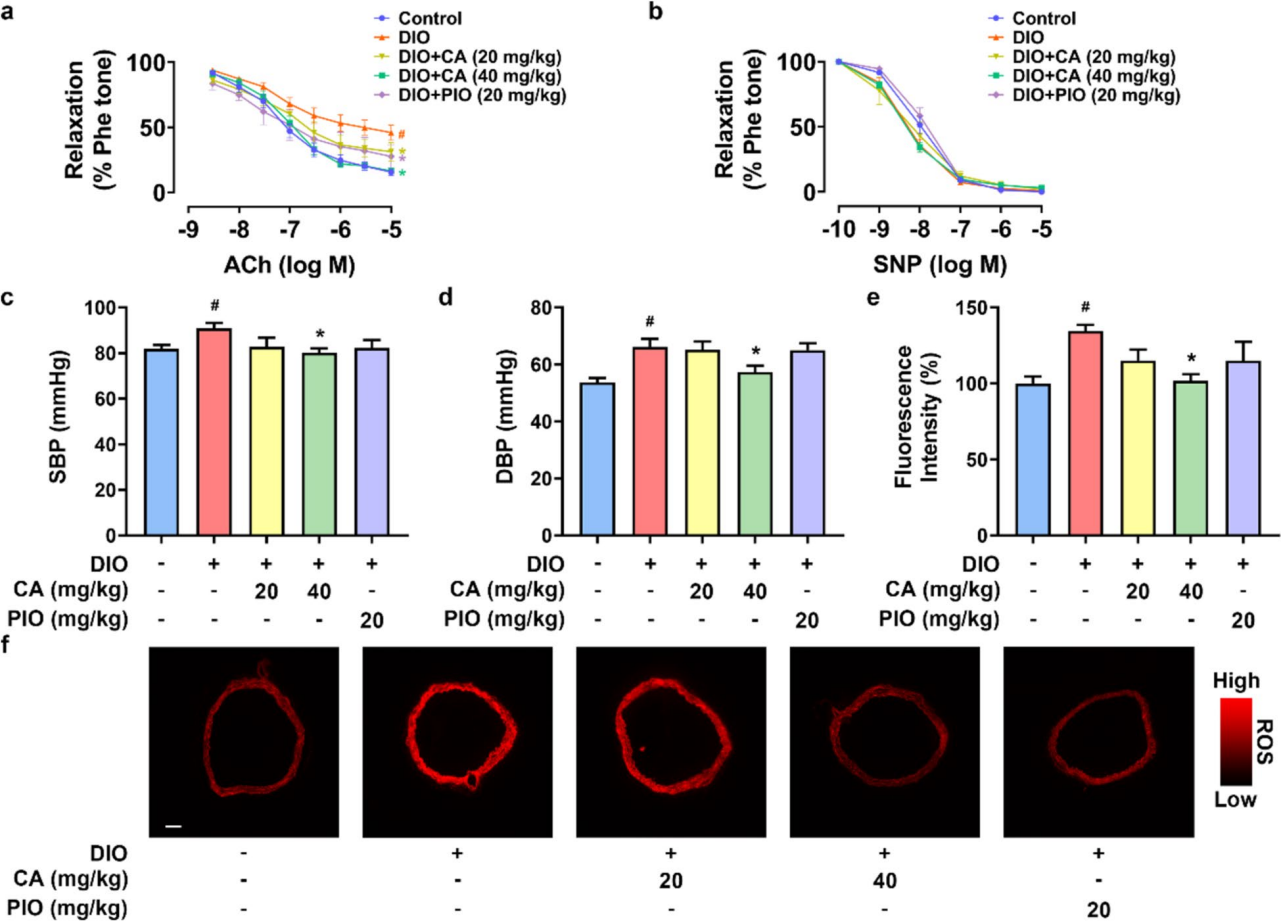
Vaso-protective effect of cinnamic acid (CA) in diet-induced obese (DIO) mice. **a** Acetylcholine (ACh)-induced endothelium-dependent relaxations (EDRs) in aortas from DIO mice with treatment of CA (20 or 40 mg/kg/day, 4 weeks) or pioglitazone (PIO, 20 mg/kg/day, 4 weeks); **b** Sodium nitroprusside (SNP)-induced endothelium-independent relaxations; **c**, **d** Changes in systolic (SBP) and diastolic blood pressures (DBP) measured by tail-cuff method; **e**, **f** Summarized data and representative images for the levels of reactive oxygen species (ROS) measured in the aortas from mice (bar, 200 μm). ^*#*^*P* < 0.05 vs. control (CON) group. ^*^P < 0.05 vs. DIO group. Data were shown as mean ± SEM (n = 6–8)

We assessed the vascular endothelial function in mouse aortas using wire myograph. The EDRs induced by ACh were impaired in the aortas from DIO mice. Four-week oral administration of CA (20 and 40 mg/kg/day) and PIO (20 mg/kg/day) significantly improved EDRs (Fig. 2a), with the high dosage of CA at 40 mg/kg/day showing more potent effect and comparable with the CON group. Meanwhile, endothelium-independent relaxations induced by SNP, reflecting the response of vascular smooth muscle to NO, were not affected among groups (Fig. 2b). The blood pressures were measured at the end of the animal study. The results showed that DIO mice had higher SBP and DBP than CON lean non-diabetic mice, whereas CA treatment at 40 mg/kg/day significantly reduced the blood pressures (Fig. 2c, d). Lower dosage of CA and PIO treatments did not significantly alter blood pressures in DIO mice. Additionally, the aortas of diabetic mice exhibited higher levels of oxidative stress when compared with the CON group, which was notably reversed after treatment with CA at 40 mg/kg/day (Fig. 2e, f).

### CA activated PPARδ and antioxidant signaling pathways in DIO mouse aortas

**Fig. 3:**
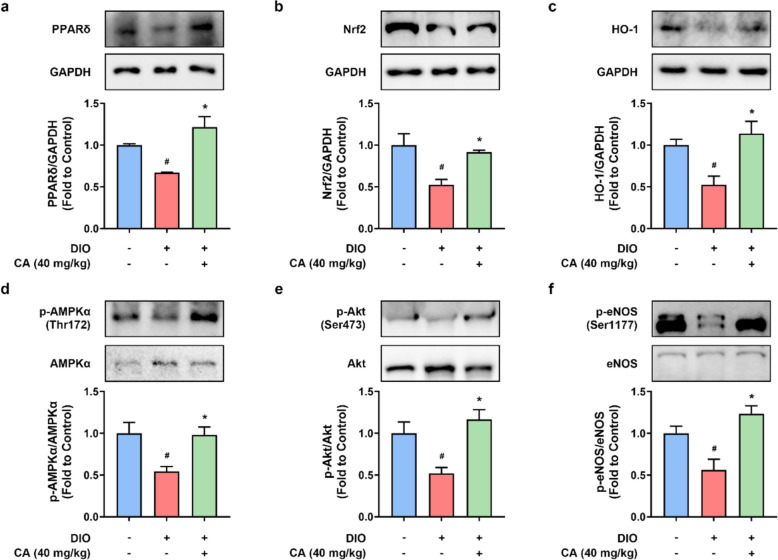
The impact of cinnamic acid on endothelial nitric oxide synthase (eNOS) and antioxidant pathways in mouse aortas. Representative bands and densitometry of the protein levels of **a** PPARδ (52 kDa), **b** Nrf2 (110 kDa), and **c** HO-1 (33 kDa) relative to GAPDH (36 kDa), **d** phosphorylated (p)-AMPKα (at Thr172) relative to total AMPKα (62 kDa), **e** p-Akt (at Ser473) relative to total Akt (60 kDa), and **f** p-eNOS (at Ser1177) relative to total eNOS (140 kDa). ^*#*^*P* < 0.05 vs. control (CON) group. ^*^P < 0.05 vs. diet-induced obese (DIO) group. Data were shown as mean ± SEM (n = 6–8)

Long-term consumption of high-fat diet resulted in the downregulation of PPARδ (Fig. 3a), and Nrf2/ HO-1, an antioxidant signaling pathway (Fig. 3b, c). These protein expressions were restored by CA treatment at 40 mg/kg/day (Fig. 2a–c), which were possibly involved in modulating the regulation of endothelial function and oxidative stress. Moreover, AMPK/Akt/eNOS signaling pathway is well known to regulate endothelial function. It was remarkably inhibited in the aortas from DIO mice. CA raised the phosphorylation levels of AMPKα at Thr172 (Fig. 3d), Akt at Ser473 (Fig. 3e) and eNOS at Ser1177 (Fig. 3f). These results demonstrated that CA activated PPARδ, Nrf2/HO-1, and AMPK/Akt/eNOS signaling pathways, which at least partially contributed to its protective effects against endothelial dysfunction and oxidative stress in obesity and diabetes.

### CA improved HG-impaired vasodilation in mouse aortas ex vivo

**Fig. 4:**
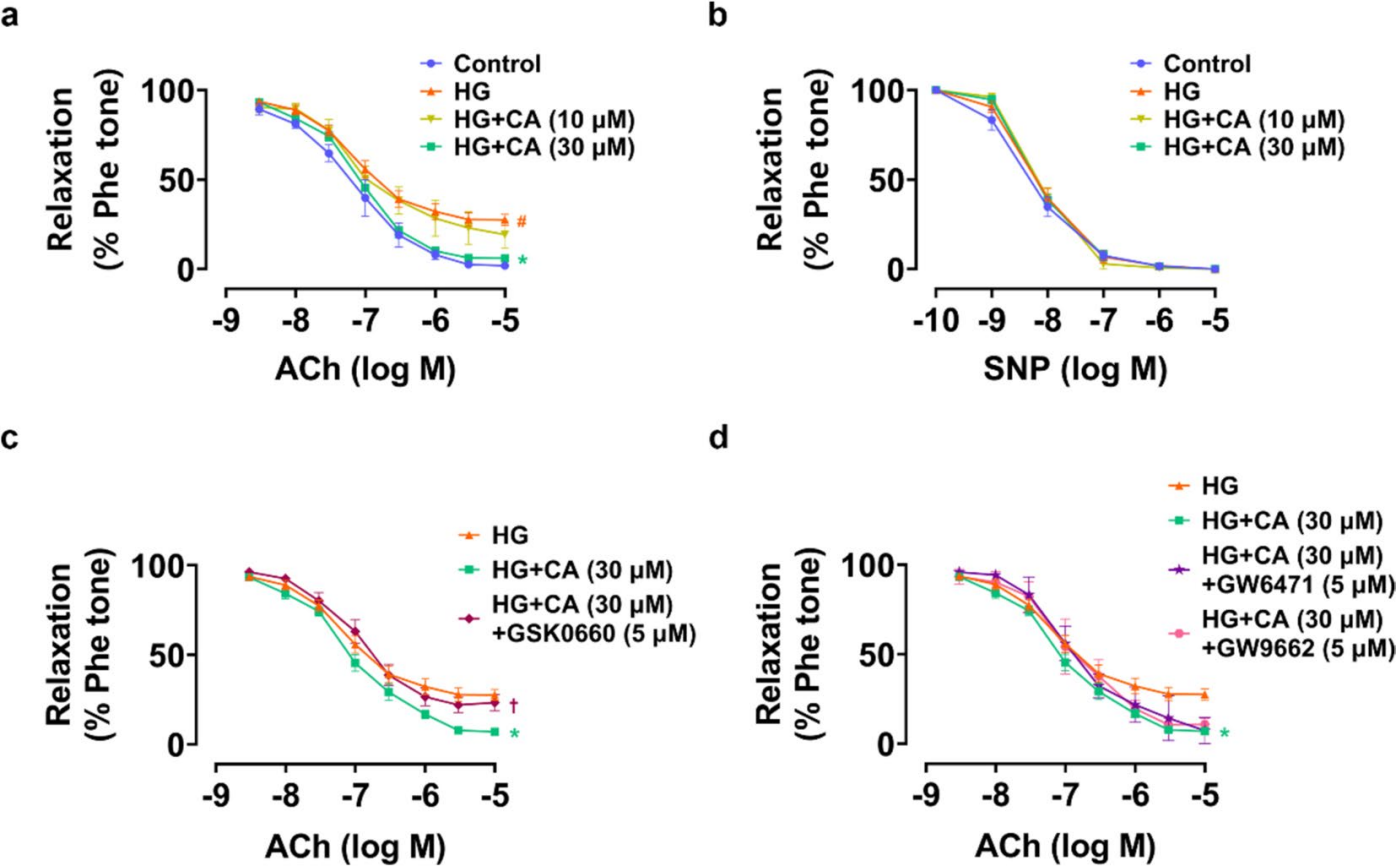
Vaso-protective effect of cinnamic acid (CA) in mouse aortas ex vivo. **a** Acetylcholine (ACh)-induced endothelium-dependent relaxations (EDRs) of aortas treated with high glucose (HG, 30 mM) and CA (10 or 30 µM) for 48 h; **b** Sodium nitroprusside (SNP)-induced relaxations; **c**, **d** Effects of PPARδ antagonist GSK0660 (5 μM), PPARα antagonist GW6471 (5 μM) and PPARγ antagonist GW9662 (5 μM) on ACh-induced EDRs in aortas that were co-treated with CA (30 μM). ^*#*^*P* < 0.05 vs. normal glucose (NG) group. ^*^P < 0.05 vs. HG group. †*P* < 0.05 vs. HG + CA (30 μM) group. Data were shown as mean ± SEM (n = 6)

We conducted functional study of mouse aortas ex vivo by wire myograph to further validate the vaso-protective effect of CA. Exposure to HG at 30 mM for 48 h disrupted EDRs in mouse aortas, and this damage was reversed by co-treatment of CA at 30 µM but not at 10 µM (Fig. 4a). Meanwhile, SNP-induced endothelium-independent relaxations were not affected (Fig. 4b). To further investigate the possible involvement of PPARs in modulating the vaso-protective effects of CA, antagonists to the three PPAR isoforms were applied. We found that only PPARδ antagonist GSK0660 (5 μM) partially inhibited the effect of CA (30 µM) on EDRs (Fig. 4c). In contrast, inhibition of the other two subtypes, PPARα by GW6471 (5 μM) and PPARγ by GW9662 (5 μM) had minimal impact on the protective effect of CA (Fig. 4d). These findings suggested that CA exerted protective role on the vascular endothelium in PPARδ-dependent manner.

### CA alleviated oxidative stress and enhanced NO production in HG-induced RAECs

**Fig. 5:**
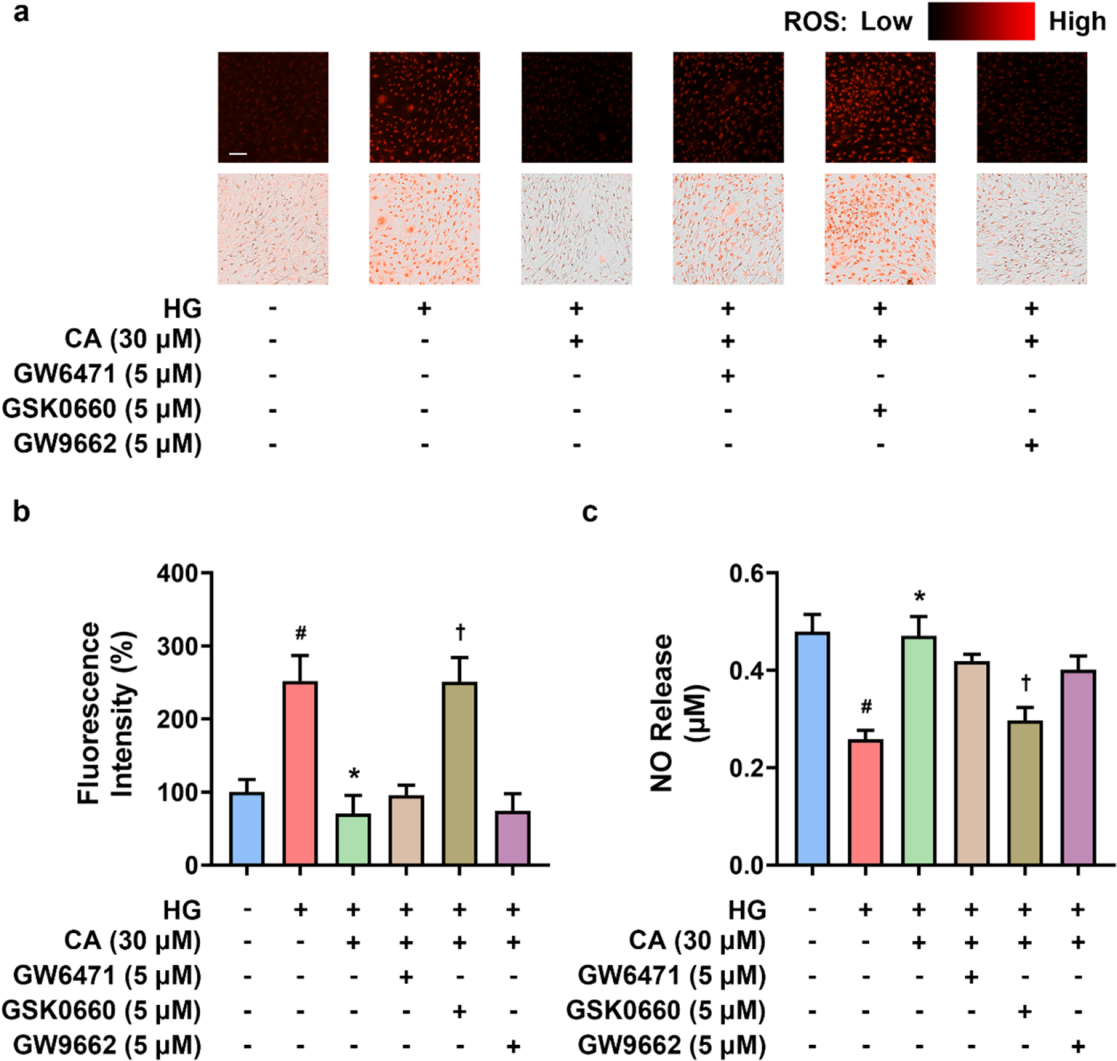
The effect of cinnamic acid (CA) on generation of reactive oxygen species (ROS) and nitric oxide (NO) in rat aortic endothelial cells (RAECs). **a**, **b** Representative images and summarized data for ROS levels measured in RAECs treated with high glucose (HG, 44.4 mM), CA (30 µM) treatment, and different PPAR antagonists (GW6471 for PPARα, GSK0660 for PPARδ, and GW9662 for PPARγ; 5 μM) for 48 h (bar, 200 μm); **c** The release of NO in the culture supernatant of RAECs examined by Griess reagents. ^*#*^*P* < 0.05 vs. normal glucose (NG) group. ^*^P < 0.05 vs. HG group. †*P* < 0.05 vs. HG + CA (30 μM) group. Data were shown as mean ± SEM (n = 4)

To confirm the effect of CA on endothelium, oxidative stress and NO production were measured in RAECs. Similarly, we detected a significant increase in the ROS levels in RAECs upon HG induction, and CA (30 μM) visibly improved this cellular damage (Fig. 5a, b). Among the three different PPAR antagonists (5 μM), only PPARδ antagonist GSK0660 showed a noticeable decrease in the efficacy of CA (Fig. 5a, b). Regarding to the critical role of eNOS/NO pathway in the regulation of vasorelaxation, we also examined the NO release into the culture supernatant of RAECs by the Griess assay. As depicted in Fig. 5c, CA recovered the decline of NO levels caused by HG treatment, whilst the effect of CA was inhibited by GSK0660. These findings indicated that among three PPAR subtypes, PPARδ was probably a crucial modulator for CA to exert protective effects in endothelial cells.

### CA activated Nrf2/HO-1 and AMPK/Akt/eNOS pathways in HG-induced RAECs

**Fig. 6:**
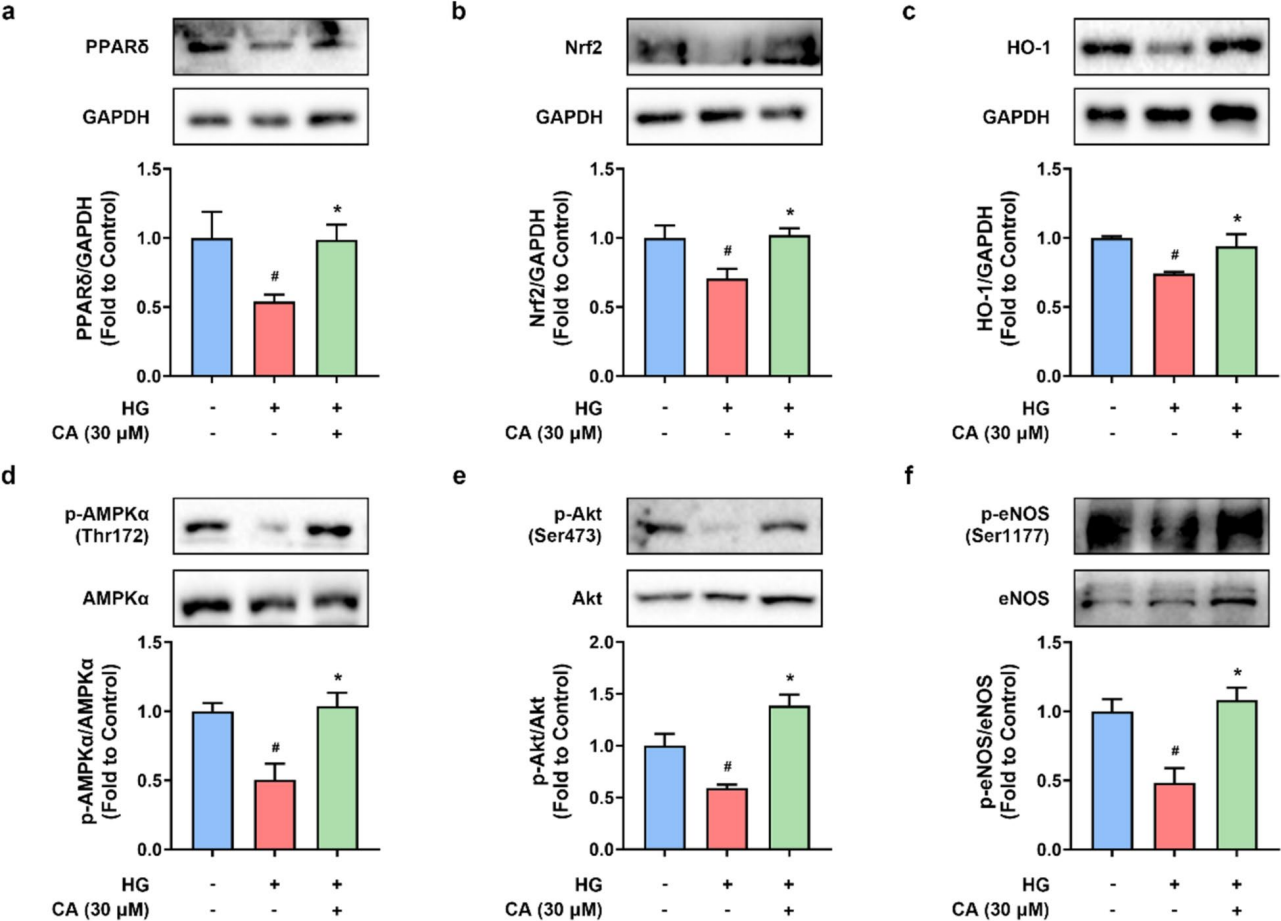
The impact of cinnamic acid on endothelial nitric oxide synthase (eNOS) and antioxidant pathways in rat aortic endothelial cells (RAECs). Representative bands and densitometry of the protein levels of **a** PPARδ (52 kDa), **b** Nrf2 (110 kDa), and **c** HO-1 (33 kDa) relative to GAPDH (36 kDa), **d** phosphorylated (p)-AMPKα (at Thr172) relative to total AMPKα (62 kDa), **e** p-Akt (at Ser473) relative to total Akt (60 kDa), and **f** p-eNOS (at Ser1177) relative to total eNOS (140 kDa) in high glucose (HG, 44.4 mM)-induced RAECs. ^*#*^*P* < 0.05 vs. normal glucose (NG) group. ^*^P < 0.05 vs. HG group. Data were shown as mean ± SEM (n = 4–6)

In line with the results from vascular tissues (Fig. 3), stimulation of RAECs with HG for 48 h suppressed the protein expressions of PPARδ, Nrf2 and HO-1 (Fig. 6a–c), as well as phosphorylation levels of AMPKα at Thr172 (Fig. 6d), Akt at Ser473 (Fig. 6e) and eNOS at Ser1177 (Fig. 6f). Co-treatment of CA at 30 µM significantly attenuated these HG-induced changes.

### CA could directly interact with PPARδ

**Fig. 7:**
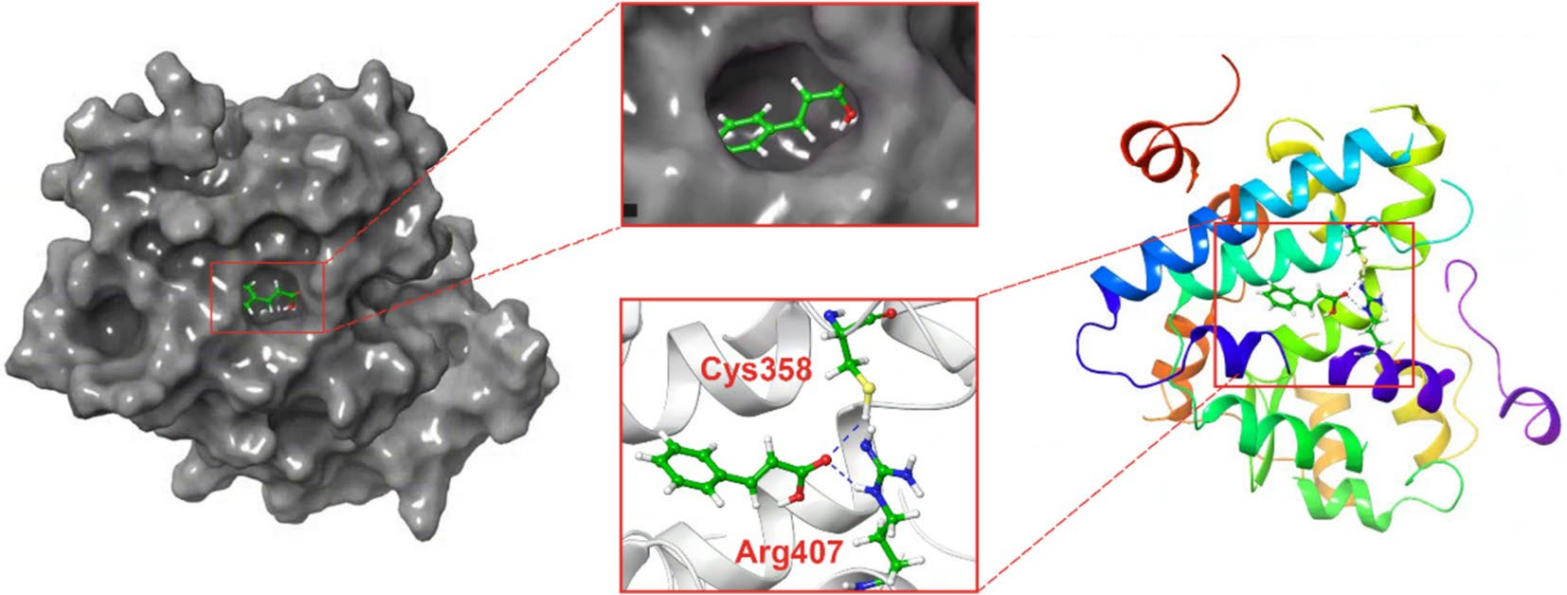
The potential binding of cinnamic acid (CA) with peroxisome proliferator-activated receptor delta (PPARδ). This figure showed the binding of CA with PPARδ through docking simulations. It also included enlarged illustrations highlighting the binding domains and sites of interaction between CA and PPARδ, respectively. Binding sites: Cys358 and Arg407 (as shown); Binding method: mainly hydrogen bonding; Binding energy: −5.77 kJ/mol

Our results showed that CA might target PPARδ to exert its pharmacological effects in vasculature. Therefore, we performed molecular docking to predict the direct interaction between CA and PPARδ. The results revealed that CA could seamlessly integrate into the structural domain of PPARδ, forming a binding pocket and establishing hydrogen bonds with the termini of two amino acids, Cys358 and Arg407 (Fig. 7). The calculated binding energy of −5.77 kJ/mol indicated a high likelihood of mutual binding between CA and PPARδ, thereby supporting the notion that CA could exert protective effects by targeting PPARδ.

## Discussion

Diabetes can significantly impact blood pressure, glucose, and lipid levels [23]. Prolonged consumption of excessive fat can lead to elevated blood glucose and lipid levels [24], exceeding healthy standards. This long-term stimulation of blood vessels can result in heightened blood pressure [25]. All these factors are closely associated with vascular injury and may cause multiple secondary cardiovascular diseases [26–28]. Therefore, blood-related indicators serve as effective markers to clearly reflect the extent of vascular damage and indirectly indicate the progression of disease within the body.

Comprehensive evidence has demonstrated that CA and its derivatives have promising therapeutic effects against diabetes [5, 29]. Sirichai Adisakwattana et al*.* reported that CA derivatives could stimulate pancreatic β-cells to secrete insulin [30]. Rahman M. Hafizur et al*.* claimed that CA could exert anti-diabetic activity by improving glucose tolerance and stimulating insulin secretion in vivo and in vitro [31]. Moreover, CA derivatives have synergistic interaction with oral hypoglycemic drugs such as thiazolidinedione and metformin, which were beneficial for the treatment of diabetes [32]. In line with these previous findings, the present study showed that oral administration of CA could reduce body weights, FBG and blood pressure and improve lipid profile in DIO mice.

Endothelial destruction can indeed contribute to the occurrence and development of multiple diabetic complications, embodying hypertension, nephropathy, atherosclerosis, and cardiomyopathy [26, 33, 34]. Therefore, targeted inhibition and reversal of endothelial injury are crucial for effective prevention and treatment of vascular complications in diabetes. Our data confirmed the induction of high blood pressure and impairment of EDRs in DIO mice; whilst CA administration reversed these impairments. Furthermore, a dose-dependent beneficial effect of CA was observed using ex vivo organ culture of mouse aortas induced with HG. Proper functioning of endothelium is closely related to oxidative stress [26] and NO bioavailability [35]. Previous study has shown the inhibitory effect of CA and its derivatives against oxidant stress [36–38]. In line with this, we found that there was an elevation of ROS level in the aortas of DIO mice, which was restored by CA treatment. Likewise, this can be observed in high glucose-stimulated endothelial cells.

Some previous studies has reported correlations between the action of CA and PPARα [39, 40] or PPARγ [41, 42], even providing evidences of CA as a ligand docking with PPARγ [43, 44]. Among the three isoforms of PPARs, there are limited findings on the possible involvement of PPARδ. One previous study from Ajay Chauhan et al*.* has suggested the potential of CA and its derivatives as PPARδ agonists [14]. To further confirm the role of PPAR in the vascular protective effects of CA, selective antagonists to the three PPAR isoforms were applied. The effects of CA to improve EDRs, NO production and oxidative stress in vasculature were blocked by the selective PPARδ antagonist GSK0660. The other two antagonists to PPARα and PPARγ were ineffective to impact the effects of CA. The molecular docking analysis also suggested a possible direct interaction between CA and PPARδ. Therefore, this suggested that CA exerted vaso-protective effects through targeting PPARδ.

Given that Nrf2/HO-1 is one of the important signaling pathway in modulating oxidative stress [45] and is linked with PPARs, we examined the effects of CA on Nrf2/HO-1 signaling pathway. Nrf2 is a transcription factor and a major redox regulator, which is participated in abundant mechanisms related to gene transcription and oxidative stress [46]. As one of the downstream effectors of Nrf2, HO-1 is a stress protease that serves as a biomarker for oxidative stress and metabolic diseases [47]. Besides, HO-1 is a downstream protein that can be modulated by PPARs [48]. The upregulation of HO-1 induced by PPARγ mediated the inflammatory response in acute lung injury/acute respiratory distress syndrome [49]. Moreover, PPARδ has been shown to protect vascular endothelial cells [50, 51] and mitigate lipid imbalance [52] associated with diabetes by regulating HO-1. Both Nrf2 and PPARs are transcription factors involved in the regulation of various cellular processes, including antioxidant responses and metabolic regulation [53–55]. In addition, there are reports implying that the inhibition or activation of PPARs could affect the functional level of Nrf2 [56, 57]. Our findings from western blot and DHE staining indicated that the protective effect of CA on diabetes-related vascular injury and oxidative stress lies in its activation of Nrf2/HO-1 pathway and PPARδ.

Compelling evidence indicates the significant involvement of AMPK/Akt/eNOS signaling in vasodilatation, particularly in conduit aortas, and with close interactions with PPARs as well as Nrf2/HO-1 signaling. NO produced by eNOS plays crucial roles in various biological functions, including the regulation of blood flow and vascular tone, metabolic activity and immune function [58]. There are various proteins modulators affecting eNOS activity and thus NO production, such as AMPK [59] and Akt signaling pathways [60]. AMPK and Akt are two important proteins that are widely implicated in physiological activities related to oxidative stress and endothelial function [61–63]. Notably, earlier work by others showed that different subtypes of PPARs could interact with AMPK and Akt, thereby mediating different physiological processes [64–66]. We also showed that CA treatment enhanced the expressions of PPARδ, Nrf2, HO-1 and activation of AMPK, Akt and eNOS in the aortas from diabetic mice and in endothelial cells, which were accompanied by decreased ROS production and increased NO release.

It is a limitation that the direct binding between CA and PPARδ is not examined in the present study, which remains to be confirmed by biochemical and biophysical methods like co-immunoprecipitation and fluorescence resonance energy transfer in the future. Further in vivo studies to utilize gene knockout mice such as PPARδ knockout mice are also required to validate the action target of CA.

In summary, the current study demonstrated an endothelial-protective effect of CA in obese diabetic mice through activating PPARδ, Nrf2/HO-1 and AMPK/Akt/eNOS signaling pathways. Thus, CA might offer preventive and therapeutic benefits in combating vascular dysfunction in obesity and diabetes. The current findings imply the prospective of the potential use of CA as health supplement or therapeutic agent for patients with diabetes and obesity, possibly counteracting the development and progression of cardiovascular and metabolic abnormalities.

## Data Availability

Not applicable.

## References

[1] Wu Y, Ding Y, Tanaka Y, Zhang W. Risk factors contributing to type 2 diabetes and recent advances in the treatment and prevention. Int J Med Sci. 2014;11(11):1185–200. 25249787 10.7150/ijms.10001 PMC4166864

[2] Shi Y, Vanhoutte PM. Macro- and microvascular endothelial dysfunction in diabetes. J Diabetes. 2017;9(5):434–49. 28044409 10.1111/1753-0407.12521

[3] Meza CA, La Favor JD, Kim DH, Hickner RC. Endothelial dysfunction: Is there a hyperglycemia-induced imbalance of NOX and NOS? Int J Mol Sci. 2019;20(15):3775. 31382355 10.3390/ijms20153775 PMC6696313

[4] Xu L, Li Y, Dai Y, Peng J. Natural products for the treatment of type 2 diabetes mellitus: Pharmacology and mechanisms. Pharmacol Res. 2018;130:451–65. 29395440 10.1016/j.phrs.2018.01.015

[5] Adisakwattana S. Cinnamic acid and its derivatives: Mechanisms for prevention and management of diabetes and its complications. Nutrients. 2017;9(2):163. 28230764 10.3390/nu9020163 PMC5331594

[6] Ruwizhi N, Aderibigbe BA. Cinnamic acid derivatives and their biological efficacy. Int J Mol Sci. 2020;21(16):5712. 32784935 10.3390/ijms21165712 PMC7460980

[7] Vogt T. Phenylpropanoid biosynthesis. Mol Plant. 2010;3(1):2–20. 20035037 10.1093/mp/ssp106

[8] Hoskins JA. The occurrence, metabolism and toxicity of cinnamic acid and related compounds. J Appl Toxicol JAT. 1984;4(6):283–92. 6394637 10.1002/jat.2550040602

[9] Wang CY, Wang SY, Chen C. Increasing antioxidant activity and reducing decay of blueberries by essential oils. J Agric Food Chem. 2008;56(10):3587–92. 18442248 10.1021/jf7037696

[10] Sun Y, Zhou L, Liao T, Liu J, Yu K, Zou L, Zhou W, Liu W. Comparing the effect of benzoic acid and cinnamic acid hydroxyl derivatives on polyphenol oxidase: activity, action mechanism, and molecular docking. J Sci Food Agric. 2022;102(9):3771–80. 34921410 10.1002/jsfa.11725

[11] Karatas O, Balci Yuce H, Taskan MM, Gevrek F, Alkan C, Isiker Kara G, Temiz C. Cinnamic acid decreases periodontal inflammation and alveolar bone loss in experimental periodontitis. J Periodontal Res. 2020;55(5):676–85. 32335913 10.1111/jre.12754

[12] Anantharaju PG, Gowda PC, Vimalambike MG, Madhunapantula SV. An overview on the role of dietary phenolics for the treatment of cancers. Nutr J. 2016;15(1):99. 27903278 10.1186/s12937-016-0217-2 PMC5131407

[13] Raha S, Paidi RK, Dutta D, Pahan K. Cinnamic acid, a natural plant compound, exhibits neuroprotection in a mouse model of Sandhoff disease via PPARalpha. NeuroImmune Pharm Ther. 2024;3(1):17–32. 38532783 10.1515/nipt-2023-0027 PMC10961485

[14] Chauhan A, Grewal AS, Pandita D, Lather V. Novel cinnamic acid derivatives as potential PPARdelta agonists for metabolic syndrome: Design, synthesis, evaluation and docking studies. Curr Drug Discov Technol. 2020;17(3):338–47. 30868955 10.2174/1570163816666190314124543

[15] Tran N, Garcia T, Aniqa M, Ali S, Ally A, Nauli SM. Endothelial nitric oxide synthase (eNOS) and the cardiovascular system: In physiology and in disease states. Am J Biomed Sci Res. 2022;15(2):153. 35072089 PMC8774925

[16] Molnar J, Yu S, Mzhavia N, Pau C, Chereshnev I, Dansky HM. Diabetes induces endothelial dysfunction but does not increase neointimal formation in high-fat diet fed C57BL/6J mice. Circ Res. 2005;96(11):1178–84. 15879311 10.1161/01.RES.0000168634.74330.ed

[17] Xue M, Qian Q, Adaikalakoteswari A, Rabbani N, Babaei-Jadidi R, Thornalley PJ. Activation of NF-E2–related factor-2 reverses biochemical dysfunction of endothelial cells induced by hyperglycemia linked to vascular disease. Diabetes. 2008;57(10):2809–17. 18633117 10.2337/db06-1003 PMC2551693

[18] Michalik L, Auwerx J, Berger JP, Chatterjee VK, Glass CK, Gonzalez FJ, Grimaldi PA, Kadowaki T, Lazar MA, O’Rahilly S, Palmer CN, Plutzky J, Reddy JK, Spiegelman BM, Staels B, Wahli W. International Union of Pharmacology. LXI. Peroxisome proliferator-activated receptors. Pharmacol Rev. 2006;58(4):726–41. 17132851 10.1124/pr.58.4.5

[19] Monsalve FA, Pyarasani RD, Delgado-Lopez F, Moore-Carrasco R. Peroxisome proliferator-activated receptor targets for the treatment of metabolic diseases. Mediators Inflamm. 2013;2013: 549627. 23781121 10.1155/2013/549627 PMC3678499

[20] Berger J, Moller DE. The mechanisms of action of PPARs. Ann Rev Med. 2002;53:409–35. 11818483 10.1146/annurev.med.53.082901.104018

[21] Cheang WS, Tian XY, Wong WT, Huang Y. The peroxisome proliferator-activated receptors in cardiovascular diseases: experimental benefits and clinical challenges. Br J Pharmacol. 2015;172(23):5512–22. 25438608 10.1111/bph.13029 PMC4667853

[22] Tian XY, Wong WT, Wang N, Lu Y, Cheang WS, Liu J, Liu L, Liu Y, Lee SS, Chen ZY, Cooke JP, Yao X, Huang Y. PPARdelta activation protects endothelial function in diabetic mice. Diabetes. 2012;61(12):3285–93. 22933110 10.2337/db12-0117 PMC3501853

[23] Reaven GM. Insulin resistance, hyperinsulinemia, hypertriglyceridemia, and hypertension: Parallels between human disease and rodent models. Diabetes Care. 1991;14(3):195–202. 2044435 10.2337/diacare.14.3.195

[24] Malone JI, Hansen BC. Does obesity cause type 2 diabetes mellitus (T2DM)? Or is it the opposite? Pediatr Diabetes. 2019;20(1):5–9. 30311716 10.1111/pedi.12787

[25] Emdin CA, Anderson SG, Woodward M, Rahimi K. usual blood pressure and risk of new-onset diabetes: Evidence from 4.1 million adults and a meta-analysis of prospective studies. J Am Coll Cardiol. 2015;66(14):1552–62. 26429079 10.1016/j.jacc.2015.07.059 PMC4595710

[26] Yang DR, Wang MY, Zhang CL, Wang Y. Endothelial dysfunction in vascular complications of diabetes: A comprehensive review of mechanisms and implications. Front Endocrinol (Lausanne). 2024;15:1359255. 38645427 10.3389/fendo.2024.1359255 PMC11026568

[27] Petrie JR, Guzik TJ, Touyz RM. Diabetes, hypertension, and cardiovascular disease: Clinical insights and vascular mechanisms. Can J Cardiol. 2018;34(5):575–84. 29459239 10.1016/j.cjca.2017.12.005 PMC5953551

[28] Yamazaki D, Hitomi H, Nishiyama A. Hypertension with diabetes mellitus complications. Hypertens Res. 2018;41(3):147–56. 29353881 10.1038/s41440-017-0008-y

[29] Zhu R, Liu H, Liu C, Wang L, Ma R, Chen B, Li L, Niu J, Fu M, Zhang D, Gao S. Cinnamaldehyde in diabetes: A review of pharmacology, pharmacokinetics and safety. Pharmacol Res. 2017;122:78–89. 28559210 10.1016/j.phrs.2017.05.019

[30] Adisakwattana S, Moonsan P, Yibchok-anun S. Insulin-releasing properties of a series of cinnamic acid derivatives in vitro and in vivo. J Agric Food Chem. 2008;56(17):7838–44. 18651742 10.1021/jf801208t

[31] Hafizur RM, Hameed A, Shukrana M, Raza SA, Chishti S, Kabir N, Siddiqui RA. Cinnamic acid exerts anti-diabetic activity by improving glucose tolerance in vivo and by stimulating insulin secretion in vitro. Phytomedicine. 2015;22(2):297–300. 25765836 10.1016/j.phymed.2015.01.003

[32] Prabhakar PK, Doble M. Interaction of cinnamic acid derivatives with commercial hypoglycemic drugs on 2-deoxyglucose uptake in 3T3-L1 adipocytes. J Agric Food Chem. 2011;59(18):9835–44. 21870829 10.1021/jf2015717

[33] Jia G, Bai H, Mather B, Hill MA, Jia G, Sowers JR. Diabetic vasculopathy: Molecular mechanisms and clinical insights. Int J Mol Sci. 2024;25(2):804. 38255878 10.3390/ijms25020804 PMC10815704

[34] Wang N, Zhang C. Oxidative stress: A culprit in the progression of diabetic kidney disease. Antioxidants (Basel). 2024;13(4):455. 38671903 10.3390/antiox13040455 PMC11047699

[35] Yuyun MF, Ng LL, Ng GA. Endothelial dysfunction, endothelial nitric oxide bioavailability, tetrahydrobiopterin, and 5-methyltetrahydrofolate in cardiovascular disease. Where are we with therapy? Microvasc Res. 2018;119:7–12. 29596860 10.1016/j.mvr.2018.03.012

[36] Nouni C, Theodosis-Nobelos P, Rekka EA. Antioxidant and hypolipidemic activities of cinnamic acid derivatives. Molecules. 2023;28(18):6732. 37764507 10.3390/molecules28186732 PMC10535275

[37] Zhuo R, Cheng X, Luo L, Yang L, Zhao Y, Zhou Y, Peng L, Jin X, Cui L, Liu F, Yang L. Cinnamic acid improved lipopolysaccharide-induced depressive-like behaviors by inhibiting neuroinflammation and oxidative stress in mice. Pharmacology. 2022;107(5–6):281–9. 35325888 10.1159/000520990

[38] Yazdi M, Nafari A, Azadpour M, Alaee M, Hadipour Moradi F, Choghakhori R, Hormozi M, Ahmadvand H. Protective effects of cinnamic acid against hyperglycemia induced oxidative stress and inflammation in HepG2 cells. Reports Biochem Mol Biol. 2023;12(1):1–12. 10.52547/rbmb.12.1.1 PMC10505459 37724158

[39] Chandra S, Roy A, Jana M, Pahan K. Cinnamic acid activates PPARalpha to stimulate Lysosomal biogenesis and lower Amyloid plaque pathology in an Alzheimer’s disease mouse model. Neurobiol Dis. 2019;124:379–95. 30578827 10.1016/j.nbd.2018.12.007 PMC6382282

[40] Prorok T, Jana M, Patel D, Pahan K. Cinnamic acid protects the nigrostriatum in a mouse model of Parkinson’s disease via peroxisome proliferator-activated receptoralpha. Neurochem Res. 2019;44(4):751–62. 30612307 10.1007/s11064-018-02705-0 PMC6450560

[41] Martinez-Rosas JR, Diaz-Torres R, Ramirez-Noguera P, Lopez-Barrera LD, Escobar-Chavez JJ, Angeles ER. PLGA nanoparticles of a new cinnamic acid derivative inhibits cellular proliferation on breast cancer cell line MCF-7 in a PPARgamma dependent way. Pharmazie. 2020;75(7):324–8. 32635974 10.1691/ph.2020.0450

[42] Miyazawa S, Sakai M, Omae Y, Ogawa Y, Shigemori H, Miyamae Y. Anti-inflammatory effect of covalent PPARgamma ligands that have a hybrid structure of GW9662 and a food-derived cinnamic acid derivative. Biosci Biotechnol Biochem. 2024;88(10):1136–43. 38944414 10.1093/bbb/zbae094

[43] Neogi P, Lakner FJ, Medicherla S, Cheng J, Dey D, Gowri M, Nag B, Sharma SD, Pickford LB, Gross C. Synthesis and structure-activity relationship studies of cinnamic acid-based novel thiazolidinedione antihyperglycemic agents. Bioorg Med Chem. 2003;11(18):4059–67. 12927868 10.1016/s0968-0896(03)00393-6

[44] Utsugi Y, Kobuchi H, Kawamura Y, Atito ASA, Nagao M, Isoda H, Miyamae Y. Importance of the proximity and orientation of ligand-linkage to the design of cinnamate-GW9662 hybrid compounds as covalent PPARgamma agonists. Molecules. 2019;24(10):2019. 31137814 10.3390/molecules24102019 PMC6571965

[45] Liu CX, Guo XY, Zhou YB, Wang H. Therapeutic role of Chinese medicine targeting Nrf2/HO-1 signaling pathway in myocardial ischemia/reperfusion injury. Chin J Integr Med. 2024;30:949–60. 38329655 10.1007/s11655-024-3657-0

[46] Liu C, Xu X, He X, Ren J, Chi M, Deng G, Li G, Nasser MI. Activation of the Nrf-2/HO-1 signalling axis can alleviate metabolic syndrome in cardiovascular disease. Ann Med. 2023;55(2):2284890. 38039549 10.1080/07853890.2023.2284890 PMC10836253

[47] Ryter SW. Heme oxygenase-1: An anti-inflammatory effector in cardiovascular, lung, and related metabolic disorders. Antioxidants (Basel). 2022;11(3):555. 35326205 10.3390/antiox11030555 PMC8944973

[48] Kronke G, Kadl A, Ikonomu E, Bluml S, Furnkranz A, Sarembock IJ, Bochkov VN, Exner M, Binder BR, Leitinger N. Expression of heme oxygenase-1 in human vascular cells is regulated by peroxisome proliferator-activated receptors. Arterioscler Thromb Vasc Biol. 2007;27(6):1276–82. 17413033 10.1161/ATVBAHA.107.142638

[49] Wang G, Han D, Zhang Y, Xie X, Wu Y, Li S, Li M. A novel hypothesis: up-regulation of HO-1 by activation of PPARgamma inhibits HMGB1-RAGE signaling pathway and ameliorates the development of ALI/ARDS. J Thorac Dis. 2013;5(5):706–10. 24255785 10.3978/j.issn.2072-1439.2013.08.69 PMC3815721

[50] Lee T, Park HS, Jeong JH, Jung TW. Kynurenic acid attenuates pro-inflammatory reactions in lipopolysaccharide-stimulated endothelial cells through the PPARdelta/HO-1-dependent pathway. Mol Cell Endocrinol. 2019;495: 110510. 31319098 10.1016/j.mce.2019.110510

[51] Ali F, Ali NS, Bauer A, Boyle JJ, Hamdulay SS, Haskard DO, Randi AM, Mason JC. PPARdelta and PGC1alpha act cooperatively to induce haem oxygenase-1 and enhance vascular endothelial cell resistance to stress. Cardiovasc Res. 2010;85(4):701–10. 19903700 10.1093/cvr/cvp365

[52] Sodhi K, Puri N, Kim DH, Hinds TD, Stechschulte LA, Favero G, Rodella L, Shapiro JI, Jude D, Abraham NG. PPARdelta binding to heme oxygenase 1 promoter prevents angiotensin II-induced adipocyte dysfunction in Goldblatt hypertensive rats. Int J Obes (Lond). 2014;38(3):456–65. 23779049 10.1038/ijo.2013.116 PMC3950586

[53] Jimenez R, Toral M, Gomez-Guzman M, Romero M, Sanchez M, Mahmoud AM, Duarte J. The role of Nrf2 signaling in PPARbeta/delta-mediated vascular protection against hyperglycemia-induced oxidative stress. Oxid Med Cell Longev. 2018;2018:5852706. 30046379 10.1155/2018/5852706 PMC6036815

[54] Abdelhamid AM, Elsheakh AR, Suddek GM, Abdelaziz RR. Telmisartan alleviates alcohol-induced liver injury by activation of PPAR-gamma/ Nrf-2 crosstalk in mice. Int Immunopharmacol. 2021;99: 107963. 34273638 10.1016/j.intimp.2021.107963

[55] Zhou B, Wang L, Yang S, Liang Y, Zhang Y, Liu X, Pan X, Li J. Pyrogallol protects against influenza A virus-triggered lethal lung injury by activating the Nrf2-PPAR-gamma-HO-1 signaling axis. MedComm (2020). 2024;5(4): e531. 38617435 10.1002/mco2.531 PMC11014464

[56] Shou JW, Li XX, Tang YS, Lim-Ho Kong B, Wu HY, Xiao MJ, Cheung CK, Shaw PC. Novel mechanistic insight on the neuroprotective effect of berberine: The role of PPARdelta for antioxidant action. Free Radic Biol Med. 2022;181:62–71. 35093536 10.1016/j.freeradbiomed.2022.01.022

[57] Barroso E, Rodriguez-Rodriguez R, Chacon MR, Maymo-Masip E, Ferrer L, Salvado L, Salmeron E, Wabistch M, Palomer X, Vendrell J, Wahli W, Vazquez-Carrera M. PPARbeta/delta ameliorates fructose-induced insulin resistance in adipocytes by preventing Nrf2 activation. Biochim Biophys Acta. 2015;1852(5):1049–58. 25728706 10.1016/j.bbadis.2015.02.010

[58] Tenopoulou M, Doulias PT. Endothelial nitric oxide synthase-derived nitric oxide in the regulation of metabolism, F1000Res 9 (2020). 10.12688/f1000research.19998.1 PMC7531049 33042519

[59] Daiber A, Xia N, Steven S, Oelze M, Hanf A, Kroller-Schon S, Munzel T, Li H. New therapeutic implications of endothelial nitric oxide synthase (eNOS) function/dysfunction in cardiovascular disease. Int J Mol Sci. 2019;20(1):187. 30621010 10.3390/ijms20010187 PMC6337296

[60] Xu J, Zou MH. Molecular insights and therapeutic targets for diabetic endothelial dysfunction. Circulation. 2009;120(13):1266–86. 19786641 10.1161/CIRCULATIONAHA.108.835223 PMC2910587

[61] Lin S, Li X, Zhang J, Zhang Y. Omentin-1: Protective impact on ischemic stroke via ameliorating atherosclerosis. Clin Chim Acta. 2021;517:31–40. 33607071 10.1016/j.cca.2021.02.004

[62] Ding Y, Zhou Y, Ling P, Feng X, Luo S, Zheng X, Little PJ, Xu S, Weng J. Metformin in cardiovascular diabetology: A focused review of its impact on endothelial function. Theranostics. 2021;11(19):9376–96. 34646376 10.7150/thno.64706 PMC8490502

[63] Zhang J, Lv W, Liu X, Sun Z, Zeng M, Kang J, Zhang Q, Liu F, Ma S, Su J, Cao K, Liu J. Ginsenoside Rh4 prevents endothelial dysfunction as a novel AMPK activator. Br J Pharmacol. 2024;181(18):3346–63. 38757416 10.1111/bph.16403

[64] Xiao L, Dong JH, Teng X, Jin S, Xue HM, Liu SY, Guo Q, Shen W, Ni XC, Wu YM. Hydrogen sulfide improves endothelial dysfunction in hypertension by activating peroxisome proliferator-activated receptor delta/endothelial nitric oxide synthase signaling. J Hypertens. 2018;36(3):651–65. 29084084 10.1097/HJH.0000000000001605

[65] Tamilmani P, Sathibabu Uddandrao VV, Chandrasekaran P, Saravanan G, Brahma Naidu P, Sengottuvelu S, Vadivukkarasi S. Linalool attenuates lipid accumulation and oxidative stress in metabolic dysfunction-associated steatotic liver disease via Sirt1/Akt/PPRA-alpha/AMPK and Nrf-2/HO-1 signaling pathways. Clin Res Hepatol Gastroenterol. 2023;47(10): 102231. 37865226 10.1016/j.clinre.2023.102231

[66] Sun Z, Tang J, You T, Zhang B, Liu Y, Liu J. LncRNA OIP5-AS1 promotes mitophagy to alleviate osteoarthritis by up-regulating PPAR-gamma to activate AMPK/Akt/mTOR pathway. Mod Rheumatol. 2024;34:1265–76. 38441253 10.1093/mr/roae015

